# CYFIP1 overexpression increases fear response in mice but does not affect social or repetitive behavioral phenotypes

**DOI:** 10.1186/s13229-019-0278-0

**Published:** 2019-06-07

**Authors:** Catherine Fricano-Kugler, Aaron Gordon, Grace Shin, Kun Gao, Jade Nguyen, Jamee Berg, Mary Starks, Daniel H. Geschwind

**Affiliations:** 1 Semel Institute, David Geffen School of Medicine, University of California, Los Angeles, Los Angeles, CA 90095 USA; 2 Department of Neurology, David Geffen School of Medicine, University of California, Los Angeles, Los Angeles, CA USA; 3 Program in Neurobehavioral Genetics, Department of Neurology, David Geffen School of Medicine, University of California, Los Angeles, Los Angeles, CA 90095 USA; 4 Center for Autism Research and Treatment, Semel Institute, David Geffen School of Medicine, University of California, Los Angeles, Los Angeles, CA 90095 USA; 5 Department of Human Genetics, David Geffen School of Medicine, University of California, Los Angeles, Los Angeles, CA 90095 USA

**Keywords:** CYFIP1, Dup15q, Autism spectrum disorder (ASD), Mouse behavior, Fear conditioning, RNA sequencing, Neurodevelopmental disorders

## Abstract

**Background:**

CYFIP1, a protein that interacts with FMRP and regulates protein synthesis and actin dynamics, is overexpressed in Dup15q syndrome as well as autism spectrum disorder (ASD). While *CYFIP1* heterozygosity has been rigorously studied due to its loss in 15q11.2 deletion, Prader-Willi and Angelman syndrome, the effects of *CYFIP1* overexpression, as is observed in patients with *CYFIP1* duplication, are less well understood.

**Methods:**

We developed and validated a mouse model of human *CYFIP1* overexpression (*CYFIP1* OE) using qPCR and western blot analysis. We performed a large battery of behavior testing on these mice, including ultrasonic vocalizations, three-chamber social assay, home-cage behavior, Y-maze, elevated plus maze, open field test, Morris water maze, fear conditioning, prepulse inhibition, and the hot plate assay. We also performed RNA sequencing and analysis on the basolateral amygdala.

**Results:**

Extensive behavioral testing in *CYFIP1* OE mice reveals no changes in the core behaviors related to ASD: social interactions and repetitive behaviors. However, we did observe mild learning deficits and an exaggerated fear response. Using RNA sequencing of the basolateral amygdala, a region associated with fear response, we observed changes in pathways related to cytoskeletal regulation, oligodendrocytes, and myelination. We also identified GABA-A subunit composition changes in basolateral amygdala neurons, which are essential components of the neural fear conditioning circuit.

**Conclusion:**

Overall, this research identifies the behavioral and molecular consequences of *CYFIP1* overexpression and how they contribute to the variable phenotype seen in Dup15q syndrome and in ASD patients with excess CYFIP1.

**Electronic supplementary material:**

The online version of this article (10.1186/s13229-019-0278-0) contains supplementary material, which is available to authorized users.

## Background

Autism spectrum disorder (ASD) is a genetically heterogenic, developmental disorder characterized by deficits in social communication as well as restrictive and repetitive behaviors. Many neurodevelopmental disorders include features of ASD, making it difficult to elucidate the underlying genetics and molecular pathways that influence ASD-associated behaviors in each distinct disorder. Copy number variations (CNVs) in the 15q11-13 chromosomal region are among the most reported genetic abnormalities in ASD [1, 2], due to multiple chromosomal breakpoints (BPs) in this region that are highly susceptible to homologous recombination [3, 4]. Duplication of 15q11-13 causes 15q duplication syndrome (Dup15q), a neurodevelopmental disorder characterized by hypotonia, developmental delay, epilepsy, and ASD [5–9]. While duplication of the BP2-3 region within 15q11-13 is sufficient to cause Dup15q, the severity of this disorder is worsened by the additional duplication of the BP1-2 region, resulting in more cognitive and behavioral problems, lower language ability, and a higher propensity to develop ASD [10, 11]. At the same time, microduplication of the BP1-2 region is associated with highly variable penetrant phenotypes ranging from neurotypical to high levels of impairment and including autistic features, language, and cognitive dysfunction [12]. Thus, analysis of genes in the BP1-2 region is of increasing interest. Here, we focus on the effect of overexpressing one of the four genes harbored in the BP1-2 region, *CYFIP1* (cytoplasmic FMRP-interacting protein 1).

In addition to its presence in BP1-2, *CYFIP1* has gained attention for its potential involvement in the etiology of Dup15q and ASD for several additional reasons: (1) It is a highly dosage sensitive gene. *CYFIP1* deletion in 15q11.2 syndrome increases risk for developmental disorders including schizophrenia [13]. (2) CYFIP1 has been shown to regulate dendritic spine formation and morphology, functioning as part of the wave regulatory complex influencing actin polymerization [14]. (3) CYFIP1 regulates protein synthesis and interacts with FMRP to regulate the translation of synaptic proteins [14, 15]. (4) Post-mortem analysis of patients with Dup15q has revealed significant overexpression of *CYFIP1* in the brain, ranging from three to 24-fold depending on the specific form of Dup15q [16, 17]. (5) In vivo and in vitro overexpression of *CYFIP1* results in abnormal neuronal morphology via dysregulation of mTOR signaling, a pathway containing many ASD-susceptibility genes [16, 18]. (6) Lastly, an analysis of *CYFIP1*’*s interactome* reveals that 19% of its associated genes are implicated in ASD and 10% in intellectual disability [15]. So, while CYFIP1 is overexpressed in ASD brain and present in the region of duplication associated with ASD, the specific contributions of *CYFIP1* overexpression on ASD-associated behaviors remain unknown.

In this study, we assess how overexpression of the highly conserved [19] human *CYFIP1* influences rodent behavior, screening specifically for deficits in social interaction, repetitive behaviors, learning and memory impairments, anxiety, and fear. Using a robust battery of behavioral testing, we determined that *CYFIP1* overexpression alone has no effect on mouse sociability and does not increase repetitive behaviors, two core behaviors in ASD. We also do not observe any increased anxiety or hyperactivity with *CYFIP1* overexpression. We do observe significant behaviors that can be comorbid with ASD and other neurodevelopmental disorders, such as transient increases in pup spontaneous vocalization [20, 21], mild learning and memory deficits [22], and, most notably, increases in conditioned fear [23]. We conducted RNA sequencing from the basolateral amygdala to take a first step towards understanding the molecular pathways that contributed to the significant increase in fear with *CYFIP1* overexpression. We found differential expression of GABA-A receptor genes, as well as genes contributing to dysregulation of neuronal plasticity, morphology, and signaling. Overall, our observations lead us to conclude that *CYFIP1* overexpression is not a major contributor to core behavioral deficits associated with Dup15q and ASD, but may affect comorbidities.

## Methods

### Generation of the CYFIP1 overexpressing mouse lines

CYFIP1-overexpressing mice were created using the UC Davis Mouse Biology Program where C57BL/6N donors received a pronuclear injection of a hCYFIP1 BAC. Founder mice with the highest hCYFIP1 expression were mated with C57BL/6N mice to produce the two lines used in this study. Genotyping was performed using 3 sets of primers:

**Table:** 

Primer name	Forward	Reverse	Sequence	DNA band size
cyfip1-595-hTgF	X		GTGAGTGGCCTCTACACCAATATGG	575 bp
cyfip1-595-hTgR		X	CCCTATTGCTGCCTTGAATTTTGG	
Cyfip1-595-3tgF	X		TCATCACAGTGACCAGGCACAGG	422 bp
Cyfip1-595-3tgR		X	GATTGATCGAATTGAGGCACTTGG	
Cyfip1-intTgF	X		GCTTGGTAGTTGTTGCACTGAAGG	286 bp
Cyfip1-intTgR		X	GGACCTAGAGTCTGAGTAGCCAAGG	

### TaqMan analysis, qPCR, and western blot

TaqMan analysis was conducted using the TaqMan Copy Number Assay (Life Technologies) using the following primers: forward: GGAGTGGAGTCCAGAGAAGAC; reverse: CATCGCTGGGAGAAATAAGCA; and probe: TGTAAACTTCCAGCTGTGCCTGC. Region dissected brain tissue (cortex, hippocampus, basolateral amydgala) from p60 mice was used for qPCR and western blot analysis. qPCR was performed using SensiFAST SYBR No-ROX kit (Bioline, Cat No. BIO-98020) and the following primers: Cyfip1 reverse: TGCTTGTTGAACCTGGTGAG; and CYFIP1 forward; ACCACATCCTGGAGACCAAG. Protein lysates were fractionated by SDS/PAGE gel and probed with anti-CYFIP1 ab (1:500, Millipore) and anti-GAPDH (1:4000, Millipore). Secondary antibody (1:5000, Millipore) conjugated to HRP was used for visualization of western blot.

### Behavioral experiments

All experiments were approved by and performed in accordance with the UCLA animal research committee. Mice had ad lib access to food and water and were group housed in a 12-h light/12-h dark cycle. All experiments were performed using male and female C57BL/6N CYFIP1 transgenic mice and their wild-type littermates. Standard N sizes for behavior range from 12 to 20 animals [24]. Many of our behavioral experiments exceed this number of animals in order to test both males and females, as well as to power us to be able to detect small, significant changes in behavior. Mice underwent multiple behavior tests. The battery of tests was designed to be performed from least stressful to most stressful in order to control the order of testing effects (Additional file 2). We waited 48 h between less stressful tests (HCB, Y-maze, and OFT) and at least 72 h between all other tests to prevent the tests from affecting one another [25].

We tested for sex effects in each behavioral task and did not find any significant results; therefore, the data is graphed with males and females combined in order to simplify the data presentation.

### SHIRPA

Details on the performance and composition of the SHIRPA screen for abnormal neurological phenotypes can be found in Irwin et al. [26]. A description of how each task was scored in this study can be found in Additional file 3: Table S2.

### Three-chambered social approach task

The three-chambered social approach task was conducted as previously described [23]. This task examines the amount of time a mouse spends with a novel mouse under a wire cup (Office Depot, item # 169990) compared to an identical, empty wire cup in another chamber. Mice that spend significantly less time with the novel mouse compared to wild-type (WT) controls are considered to have social impairments. Briefly, on the day of testing, stimulus mice were habituated to the wire cups in the arena for 10 min each in both the left and right cups prior to the test. Each test mouse was then placed in the center of an interconnected three-chambered box measuring 59.5 cm × 49.9 cm × 25.4 cm (length × width × depth) after habituation. The center chamber was empty, while left and right chambers contained an empty wire cup or a sex- and weight-matched, novel wild-type mouse in a similar wire cup. Behavior was recorded by the automated system, Top Scan (Clever Sys, Inc., Reston, VA, USA) over a 10-min period. Time spent sniffing the mouse-containing cup or the empty cup and time in each chamber were scored manually, blinded to genotype. A two-way ANOVA was used to analyze the difference between social WT and Tg social sniff accounting for the two factors of sex and genotype (Fig. 2b).

### Ultrasonic vocalizations (USVs)

This task measures the number of calls emitted from a pup calling to its mother. It is used to assess early deficits in communication. Pups were separated from their mother and placed in a soundproof box with a microphone. USVs were recorded for 5 min before the pup was returned to its home cage. USVs were processed and characterized according to previous methods [27].

### Home-cage behavior

Home-cage behavior was conducted as previously described [41]. This task is used to screen for potential repetitive behaviors-specifically excessive grooming and digging that occur in non-stressful conditions at baseline. Mice were placed in juxtaposed cages containing fresh bedding separated by opaque panels to prevent mice from observing each other. Behavior was recorded by the automated system, “CaptureStar” (Clever Sys, Inc., Reston, VA, USA), over a 20-min period. Only the last 10 min of the video were watched and then manually scored for digging and grooming. Digging and grooming were analyzed using two-way ANOVAs including sex and genotype as factors (Fig. 2d and e).

### Y-maze

This task is used to screen for repetitive behaviors. Mice were placed in a 3-armed Y-maze for 8 min and allowed to explore freely. Each of the 3 arms is 18-cm long, 11.4-cm wide, and 19.7-cm deep. The order of entries into each arm was manually recorded. A successful alternation was recorded as visiting each of the 3 arms in succession without returning the arm that the mouse had just previously visited. A mouse with an increase in repetitive behaviors will alternate less than a WT control mouse. The percentage of no alternation and number of arms visited were both analyzed using two-way ANOVAs with sex and genotype as factors (Fig. 2 f and g).

### Elevated plus maze

This task measures anxiety by analyzing how long mice spend in open, vulnerable arms of a maze compared to closed, protected arms. Mice were placed in the center of the elevated plus maze consisting of two, open, opposite arms and two, enclosed, opposite arms. Each arm is 28 cm long and 7.62 cm wide. The closed arm’s walls are 16.5 cm high and the center square which is not scored is 8 cm × 8 cm. Mice were recorded for 5 min and analyzed for the total time spent in the open and closed arms. The amount of time spent in the open versus the closed arm was analyzed using a three-way ANOVA including sex and genotype as factors (Fig. 3a). “Open” and “closed” are considered two independent measures because time spent in the center of the maze is not scored.

### Open field test

The open field test was conducted as previously described [28]. This task is used to record the distance traveled in the field over time to assess hyperactivity, as well as the amount of time a mouse spends in the center the field versus the edges. A mouse that spends more time near the edges of the arena is considered to have an increase in anxiety. Mice were placed inside of a 27.5 cm × 27.5 cm clear plexiglass arena and recorded by the automated system, TopScan (Clever Sys, Inc., Reston, VA, USA), over a 20-min period. The middle, 66% of the arena, was considered the “center” with the area outside this square considered the “surround”. The amount of time spent in the surround of the open field was analyzed using a two-way ANOVA with sex and genotype as factors (Fig. 3b). The distance traveled was analyzed by summing the distance over time to determine the total distance and then analyzing the difference using a two-way ANOVA with sex and genotype as factors (3C).

### Morris water maze

The Morris water maze is used to assess learning and memory by training mice to find a hidden platform, and measuring the amount of time it takes them to locate it. Visual cues in the form of different shapes were placed on the walls in the maze room for the mouse to use for navigation. On the first day of training, mice were acclimated to the Morris water maze (measuring 67.8 cm in diameter) by placing them on the platform for a total of 30 s, replacing them whenever they left the platform. After acclimation, mice underwent 4 training trials a day for 5 days. For each training trial, mice were placed in a random quadrant of the maze and the latency to finding the platform was recorded. Each trial ended either when the mouse successfully found the platform or after 1 min. On day 5, 1 h after training, the platform was removed for the probe trial. Mice were recorded for 60 s and assessed for how long they spent in each quadrant searching for the platform. On day 6, the platform was moved to the opposite quadrant (reversal) and mice were again trained for 5 days. On day 10, the platform was again removed for the second probe trial and time spent in each quadrant was recorded for 60-s. All recordings were analyzed using TopScan (Clever Sys, Inc., Reston, VA). Latency to platform was analyzed using two-way repeated measures ANOVA for the first 5 days and again for the reversal trials (days 6–10) with sex and genotype as factors (Fig. 3e). The time spent in each quadrant for the probe trials was analyzed using a three-way ANOVA with sex and genotype as factors (Fig. 3f and g).

### Fear conditioning

Fear conditioning is used to assess learned fear in mice by observing the amount of time they freeze when exposed to an adverse stimulus that has been paired with an auditory cue. This freezing is automatically scored by using a video camera and the software “Video Freeze” (Med Associates Inc.). Auditory fear conditioning was performed as previously described [29] with modifications. On day 1, tone/shock acquisition was performed using a 2-s, 0.5-mA shock paired with a 30-s, 80-dB tone at 2000 Hz. The tone-shock protocol consisted of a 2-min habituation followed by a 30-s tone with a 2-s shock during the last 2 s of tone. After the shock, there was a 1-min wait followed by the next 30-s tone with 2-s shock during the last 2 s of tone. After the second shock, there was one more 1-min wait, followed by a 30-s tone with 2-s shock during the last 2 s of tone, and a final 2-min wait. Freezing was recorded during the entire protocol. On day 2, the mouse was returned to the shock context for 8 min and analyzed for freezing. There were no tones or shocks during this time. On day 3, mice were placed in a novel context with no tones or shocks and their freezing was recorded for 8 min. On day 4, mice were returned to the novel context and freezing was assessed to the 80-db tone. The tone protocol consisted of a 2-min wait, 30-s tone, 1-min wait, 30-s tone, 1-min wait, 30-s tone, and 2-min wait. Freezing for day 1’s acquisition training was analyzed using a two-way repeated measures ANOVA with sex and genotype as factors (Fig. 4b). Freezing in the novel and shock contexts, as well as to the tone and during the intertone interval, were analyzed using two-way ANOVAs with sex and genotype as factors (Fig. 4c and d).

### Hot plate sensation assay

This assay is used to determine if mice have an increase in sensory processing or pain reception by exposing them to heat and recording how long it takes to react to this stimulus. Mice were first habituated to the hot plate before it was turned on by individually placing each mouse on the plate for 30 s. After all the mice had been habituated, the hot plate was turned on and heated to a constant temperature of 52.5 °C. Then, mice were individually placed on the hotplate within a 20-cm-diameter Plexiglas cylinder on all four feet and the latency to either lick the hind paws or jump with all 4 ft leaving the hotplate was recorded to the nearest 0.1 s. Latency to reflex was analyzed using a two-way ANOVA with sex and genotype as factors (Fig. 4e).

### Prepulse inhibition protocol

This assay is used to measure sensory-motor gating. Mice were placed in a restraint tube mounted on a startle measurement platform. To determine the degree of prepulse inhibition (PPI), the startle-eliciting stimulus (120-dB sound) was preceded by a brief low-intensity stimulus of 70 dB, 75 dB, or 80 dB and the new startle response at each intensity level was measured. The specific pattern of pulse and prepulse sounds used and the PPI calculation formula have been previously described [30]. Percent PPI was analyzed using a two-way repeated measures ANOVA with sex and genotype as factors (Fig. 4f).

### Protein and RNA

Mice were anesthetized at p60 with isoflurane and euthanized via decapitation. The brains were quickly removed and dissected at 4 °C where a large piece of cortex and the hippocampus was collected and flash frozen on dry ice. Dissections from the right hemisphere were reserved for RNA extraction, and the left hemisphere for protein analysis. For the BLA dissections, the fresh brain was sliced coronally at bregma and placed in a brain mold. A 1-mm slice was extracted from − 1 mm to − 3 mm of bregma. This slice was then visually inspected for the white matter tract surrounding the BLA and a 1-mm punch was used to dissect out this region. The left and right BLA were pooled for downstream processing. RNA extraction was performed using the RNeasy Mini Kit (Qiagen). Protein extraction was done using a protein lysis buffer (final concentration of 0.5 M NaCl, 0.2 mM NaVO4, 100 mM NaF, 2 mM DTT, 2 mM PMSF in Isopropanol, Protease Inhibitor tablet) and combined 1:1 with 2X lamelli buffer (biorad) for western blotting. Thirty-five out of the 142 mice used for protein and RNA analysis were also involved in behavioral testing, specifically USVs and home-cage behavior.

### RNA sequencing

RNA sequencing was performed using tissue from both transgenic lines including males and females. RNA samples were randomized and pooled for library preparation. Sample library preparation was done using Lexogen QuantSeq 3′ Fwd with pre-normalized moderate to high quality RNA and sequenced at a read depth of 10 million reads using Quant seq. Reads were mapped with STAR [31] to GRCm38 using Gencode v11. Alignment, GC bias, and duplication metrics were collected using Picard tools (http://broadinstitute.github.io/picard/) functions CollectRnaSeqMetrics, CollectGcBiasMetrics, and MarkDuplicates and were aggregated using MultiQC [32], and gene expression was quantified using Salmon [33]. Genes with less than 10 reads in over 80% of the samples were removed. Outlying samples with standardized sample network connectivity *Z*-scores < − 2 [34] were removed.

### Differential gene expression

Differential gene expression was performed using DEseq2. Differentially expressed genes were defined as having *p* value < 0.005, a threshold that we and others have previously validated [35, 36]. The models used included the following covariates: Sex + Line + SeqPC1-5 + Genotype. SeqPC1-5 are the first 5 principal components calculated from the Picard sequencing statistics and are used to control for sequencing technical variation.

### GO term enrichment

GO term enrichment was performed using GO-Elite [37] and EnsMart77Plus with default settings and 10,000 permutations. All expressed genes were used as the background set. Gene sets with less than 50 genes or which did not overlap with at least 4 genes in the test list were dropped. The top biological process and molecular function categories ranked by *Z*-score were plotted.

### Weighted gene co-expression network analysis

A gene co-expression network was constructed using the WGCNA package [38] in R after regressing out the covariates used in the differential expression model. A soft threshold was chosen to attain approximate scale-free topology (*R*^2^ > 0.8) of the network. The network was constructed using a topological overlap dissimilarity matrix. Modules were defined as using the hybrid dynamic tree-cutting method [39] on a dendogram created by hierarchical clustering.

Genotype was linked to modules using a linear model. Module hub genes were defined as being highly correlated to the module eigen gene (kME > 0.7). Cell type enrichment was performed using fisher exact test using cell type-specific genes [40].

### Statistical analysis

The software Graph Pad Prism was used to analyze data and obtain *p* values. Significance was determined using a Student’s *t* test unless otherwise noted in the figure legend. Data is presented as mean ± SEM, represented by the error bars. Specific statistical information for each figure is also noted in Additional file 3: Table S2.

## Results

### Creation and characterization of CYFIP-overexpressing mice

**Fig. 1:**
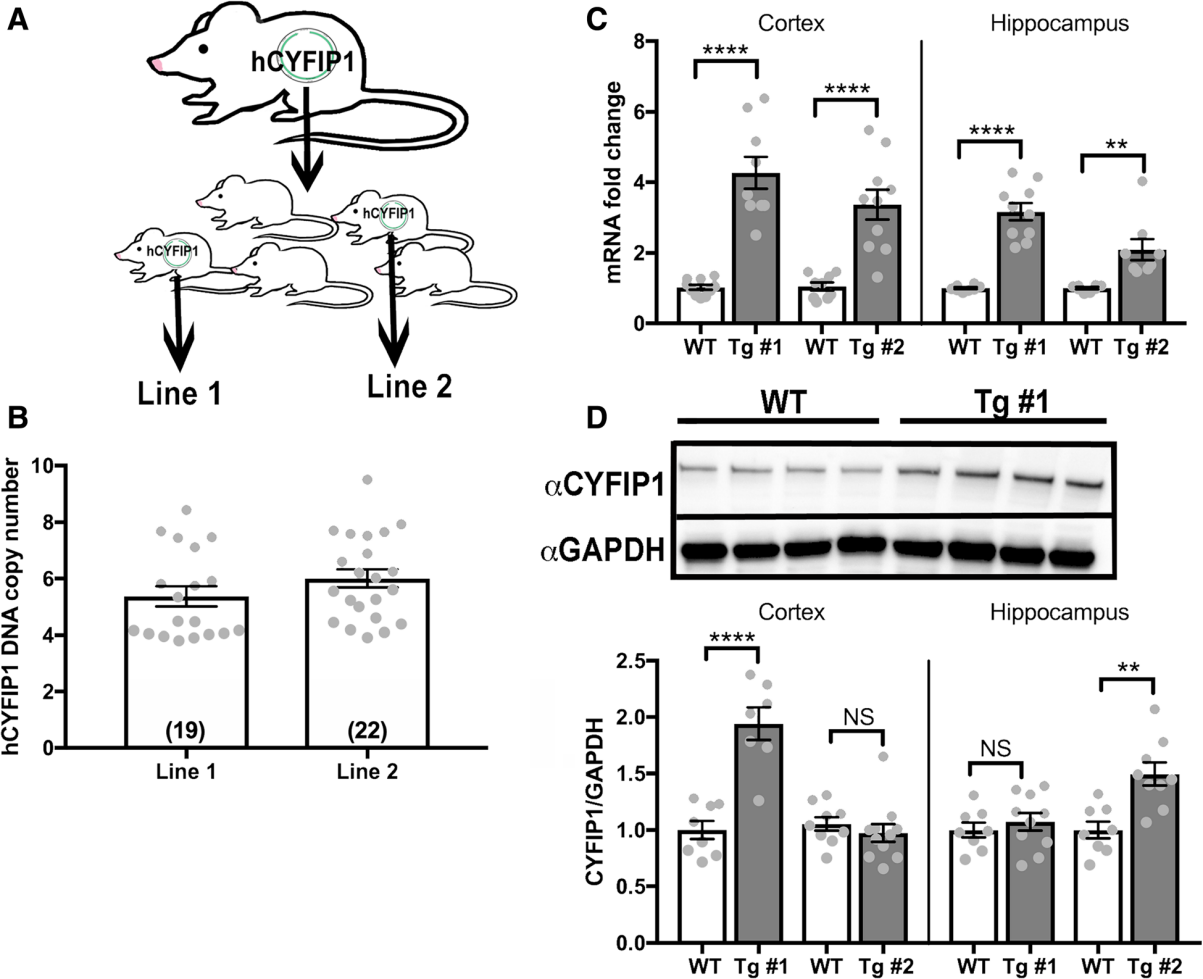
Creation and molecular characterization of human CYFIP1-overexpressing mice. **a** CYFIP1-overexpressing mice were created via pronuclear injection of a BAC containing the coding sequence for the human *CYFIP1* gene. Two progeny with the highest cDNA copy number were selected to breed with wild-type C57BL6/N mice in order to establish transgenic lines 1 and 2. **b** h*CYFIP1* copy number was confirmed using a Taqman copy number qPCR assay (Line 1 = 5.4 ± 0.34, Line 2 = 6.0 ± 0.32). **c** CYFIP1 mRNA expression was assessed in the cortex and hippocampus of p60 transgenic mice using qPCR. There was a significant increase in CYFIP1 mRNA expression in transgenic mice compared to their wild-type littermates (Line 1 cortex: WT = 1.03 ± 0.07, Tg #1 = 4.27 ± 0.45. Line 2 cortex: WT = 1.05 ± 0.11, Tg #2 = 3.37 ± 0.42. Line 1 hippocampus: WT = 1.00 ± 0.03, Tg #1 = 3.17 ± 0.24. Line 2 hippocampus: WT = 1.00 ± 0.03, Tg #2 = 2.10 ± 0.30). **d** CYFIP1 protein expression varied by region and line in p60 mice as shown by western blot analysis. The representative blot shows cortical CYFIP1 protein expression from four Line 1 WT and 4 Tg #1 animal. Line 1 had a significant increase in CYFIP1 protein expression in the cortex, but there was no detectable overexpression in the hippocampus. Line 2 showed significant CYFIP1 overexpression in the cortex but not in the hippocampus (Line 1 cortex: WT = 1.00 ± 0.80, Tg #1 = 1.94 ± 0.15. Line 2 cortex: WT = 1.05 ± 0.06, Tg #2 = 0.97 ± 0.08. Line 1 hippocampus: WT = 1 ± 0.07, Tg #1 = 1.07 ± 0.08. Line 2 hippocampus: WT = 1.00 ± 0.08, Tg #2 = 1.50 ± 0.10). *P* values were calculated using Student’s *t* test. NS, not significant. **p < 0.01. **** *p* < 0.0001. See Table S1 for *n*’s

To characterize the consequences of *CYFIP1* overexpression on mouse behavior, we utilized the Mouse Biology Program at UC Davis to create *CYFIP1*-overexpressing transgenic (Tg) mouse lines using pronuclear injection of a human-*CYFIP1* (h*CYFIP1*) bacterial artificial chromosome (BAC) (Fig. 1a). To control for insertional effects, we derived two h*CYFIP1*-overepressing Tg mouse lines, which we called line 1 and line 2. The average copy numbers for the h*CYFIP1* BAC in line 1 and line 2 were 5.4 and 6.0, respectively (Fig. 1b). mRNA transcripts for h*CYFIP1* at p60 were significantly increased in the cortex and hippocampus in both lines compared to their wild-type (WT) littermates (Fig. 1c, Additional file 3: Table S1). At p60, there was also an increase in CYFIP1 protein expression in the cortex, but not in the hippocampus of line 1 mice, while line 2 showed an increase in CYFIP1 protein in the hippocampus, but not in the cortex (Additional file 1: Figure S1D, Additional file 3: Table S1). At p21, there is a significant increase in CYFIP1 expression in the cortex in both transgenic lines, confirming overexpression, while showing that protein expression of CYFIP1 may be differentially regulated in different regions at different time points (Additional file 1: Figure S1).

### CYFIP1-overexpressing mice show no deficits in core ASD-related behaviors

We next assessed whether h*CYFIP1* mice presented with ASD-associated behaviors by performing a battery of behavioral assays. First, we first carefully examined the Tg lines for off-target BAC insertional effects using a modified SHIRPA screen [26] (Additional file 3: Table S2), which is a comprehensive, standardized diagnostic assay that screens for behavioral, neurological, and physiological abnormalities. We found no observed deficits in general health, reflexes, motor, or sensorimotor functions in either Tg line (Additional file 3: Table S2). Therefore, we assessed for abnormalities in ultrasonic vocalizations (USVs), social interactions, and repetitive behaviors in both male and female animals from multiple litters to ensure sufficient power (Additional file 3: Table S3) using protocols that have robustly identified abnormalities in other mouse models of ASD in our lab [28, 41, 42], and others [20, 22, 23].

**Fig. 2:**
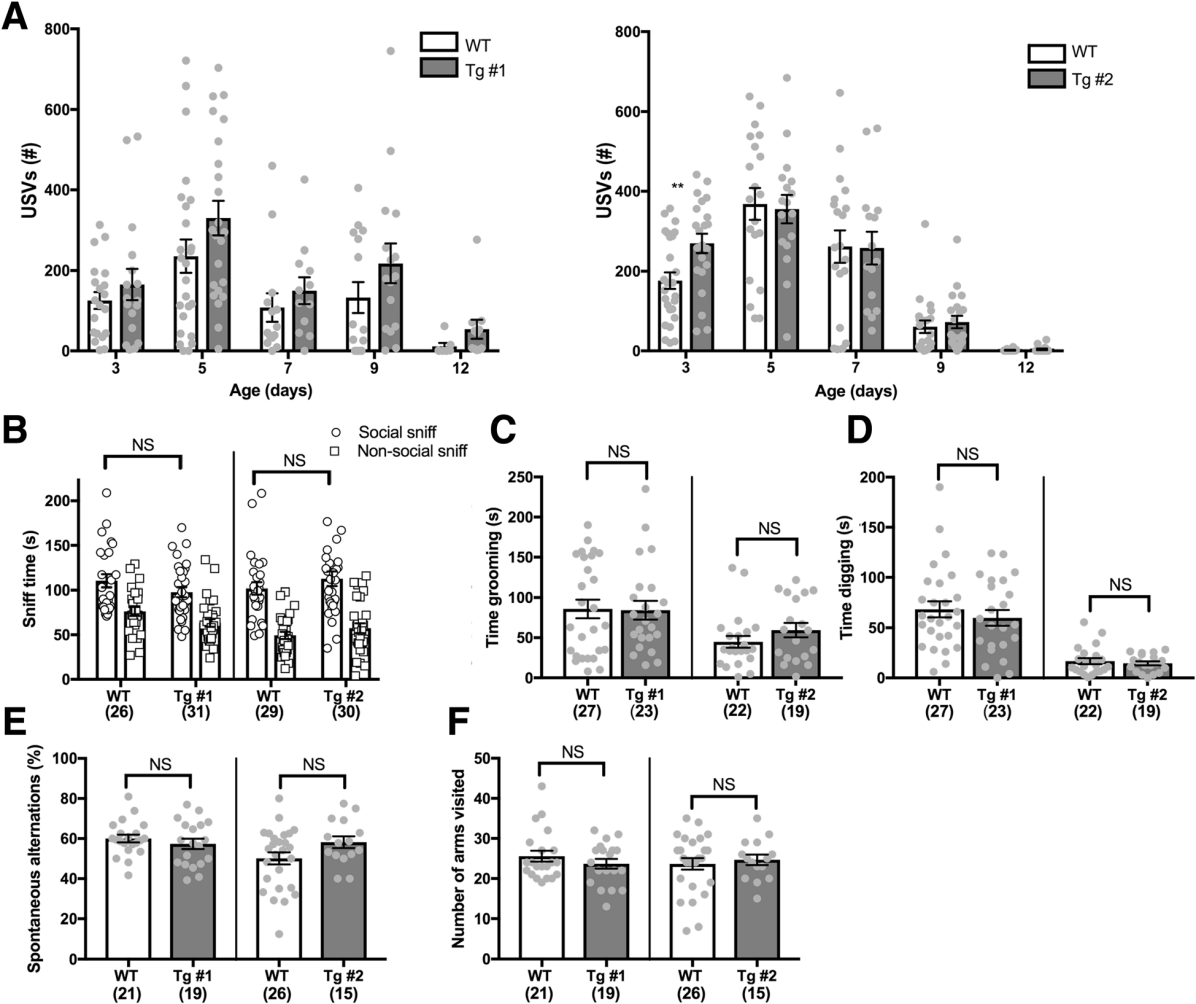
CYFIP1-overexpressing mice have no deficits in social communication or repetitive behaviors. **a** Ultrasonic vocalizations (USVs) provoked by pup/dam separation were assessed in pups at postnatal days 3, 5, 7, 9, and 12 for 5 min at each time point. There was a significant increase in the number of vocalizations at day 3 in Tg line #2 compared to their WT littermates (WT = 176.6 ± 20.8, Tg #2 = 269.3 ± 24.2). **b** CYFIP1 mice were tested for social deficits using the three-chamber social test. Both WT and Tg mice spent more time interacting with another mouse when given the choice between a stimulus mouse and an empty cup (social sniff: Line 1: WT = 110.4 ± 7.4, Tg #1 = 97.719 ± 5.5. Non-social sniff: Line 1 WT: 76.2 ± 5.1, Tg #1 = 63.2 ± 4.3. Social Sniff: Line 2: WT = 101.8 ± 6.9, Tg #2112.6 ± 8.3. Non-social sniff, WT = 49.3 ± 4.0, Tg #2, 57.4 ± 5.6). **c**, **d** Repetitive behaviors were assessed by scoring time spent grooming (**c**) and digging (**d**) over a 10-min period. No excessive grooming or digging was noted in CYFIP1 Tg mice (Grooming: Line 1: WT = 85.85 ± 11.48, Tg #1 = 84.35 ± 11.65. Line 2: WT = 44.85 ± 7.294, Tg #2 = 59.51 ± 8.97. Digging: Line 1: WT = 68.41 ± 7.997, Tg #1: 59.96 ± 7.846. Line 2: WT = 16.78 ± 2.991, Tg # 2 = 14.63 ± 2.003). **e**, **f** Mice were placed in a Y-maze and allowed to freely explore the arms for 8 min. **e** There was no significant difference in spontaneous alternations in either line (Line 1: WT = 60.05 ± 1.889, Tg #1, 57.36 ± 2.595. Line 2: WT = 50.12 ± 3.03, Tg #2, 58.22 ± 2.919). **f** There was no difference in the number of arms visited (Line 1: WT = 25.57 ± 1.362, Tg #1: 23.68 ± 1.224, Line 2: WT = 23.65 ± 1.435, Tg #2: 24.67 ± 1.315. NS, not significant. *p < 0.05. **p < 0.01. ***p < 0.001. ****p < 0.0001. See Table S3 for *n*’s and exact statistical tests

USVs were recorded in pups along a developmental trajectory from postnatal day 3 (P3) to P12 and analyzed for the number of calls recorded over 5 min [27]. There was a significant increase in the number of calls in Tg #2 mice at P3, and a similar, but non-significant, trend at the other time points, which may be indicative of increased stress [43] in these animals compared to their WT littermates (Fig. 2a). However, while trending, there was no increase in vocalization at any of the other tested ages, nor in Tg #1. Since we do not identify a significant USV phenotype in both lines or at multiple time points between P3 and P10 as is typical [44, 45], we conclude that there is no substantial change in USVs due to CYFIP1 overexpression.

Social deficits are one of the defining features of children with ASD; therefore, we utilized the widely used three-chamber test [23, 44, 46] to assess abnormalities in general sociability of CYFIP1 Tg mice. We documented the amount of time each mouse spent sniffing a novel mouse under a wire cup (social sniff) versus sniffing an empty wire cup (non-social sniff), and tested multiple litters to obtain sufficient power to identify small changes ([24], the “Methods” section). Both control and Tg mice preferred sniffing the cup containing the mouse, indicating no avoidance of social interactions (Fig. 2b). Overall h*CYFIP1* Tg mice show no deficits in social interaction as assessed by the three-chamber social test.

Restrictive, repetitive behaviors are often observed in children with ASD and considered part of the core symptoms of ASD. We assessed hCYFIP1 Tg mice for repetitive behaviors by examining them for excessive grooming or digging as has been observed in other genetic models of ASD [28, 41, 47]. There was no increase in grooming (Fig. 2c) or digging (Fig. 2d) in either Tg line compared to WT controls, indicating normal home-cage behavior. We also assessed repetitive and restrictive behaviors using the Y-maze, where control mice are expected to naturally alternate as part of their exploratory strategy [48]. The order of entries into each arm was recorded and analyzed for spontaneous alternations. Both WT and Tg mice had similar spontaneous alternation percentages (Fig. 2e) and visited a comparable number of arms (Fig. 2f), indicative of normal levels of motor activity and no perseveration.

### CYFIP1-overexpressing mice show no increases in anxiety and hyperactivity

**Fig. 3:**
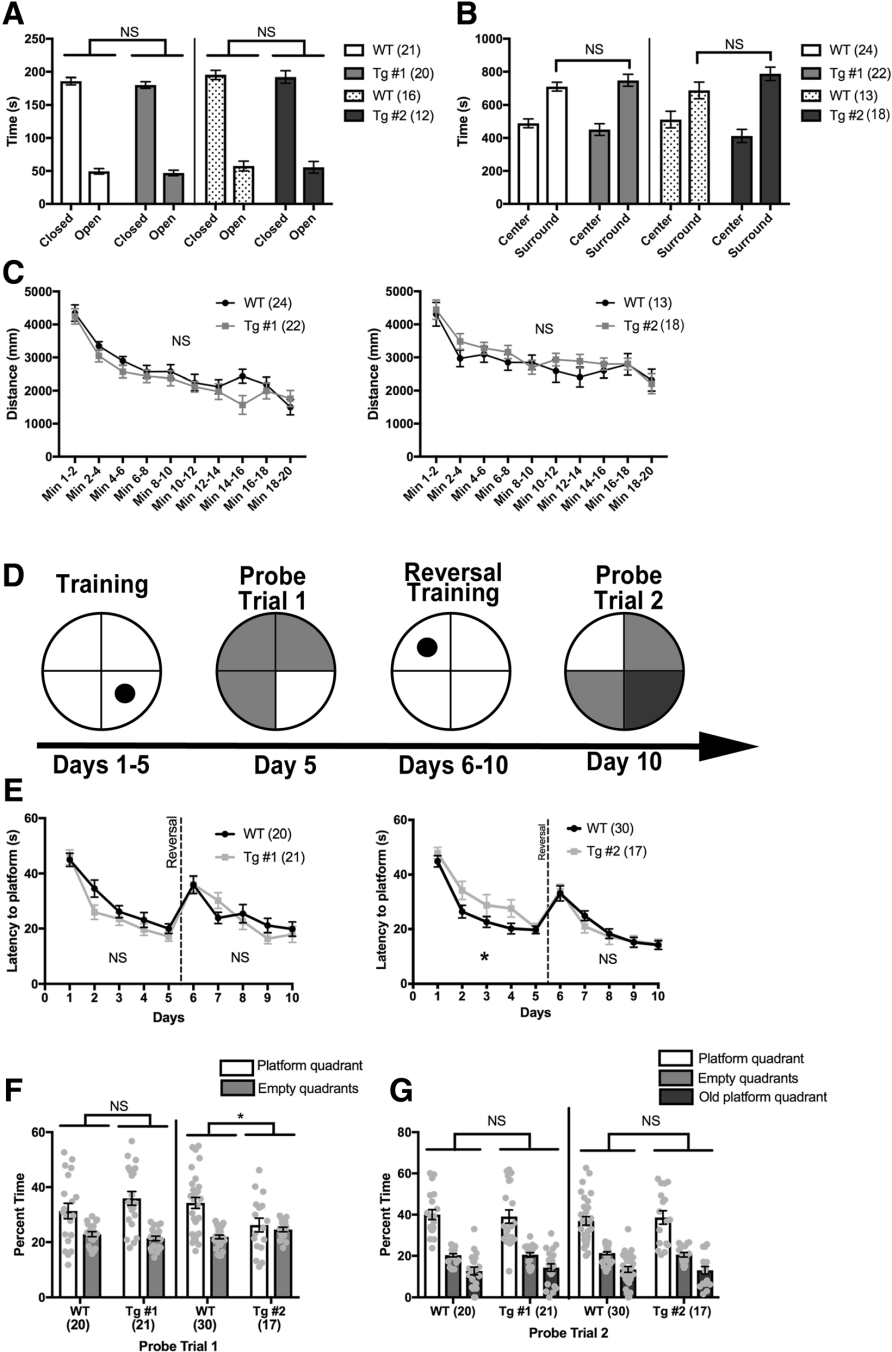
CYFIP1-overexpressing mice show no anxiety, hyperactivity, or intellectual disability. **a** Anxiety was assessed in the elevated plus maze by calculating time spent in the open arms of the maze compared to the closed arms. Tg mice the same amounts of time in the open and closed arms as their WT littermates (Line 1: WT closed arm = 186.103 ± 5.485. WT open arm = 49.5 ± 4.101. Tg #1 closed arm = 180.326 ± 4.937, Tg #1 open arm = 47.011 ± 4.224. Line 2: WT closed arm = 195.417 ± 6.695. WT open arm = 57.506 ± 7.440. Tg #2 closed arm = 192.243 ± 9.277. Tg #2 open arm = 55.541 ± 8.862. **b** Anxiety was also assessed using the open field test. There was no increase in time spent near the edges of the field (surround) in the Tg mice compared to their WT littermates, demonstrating no increased anxiety mice (Line 1: WT center = 488.575 ± 26.44, surround = 711.425 ± 26.44. Tg #1 center = 450.895 ± 35.195, surround = 749.105 ± 35.195. Line 2: WT center = 51.344 ± 50.432, surround = 688.656 ± 50.432. Tg #2 center: = 411.978 ± 40.114, surround = 788.022 ± 40.114). **c** Hyperactivity was assessed using the open field test by measuring the distance traveled over 20 min. There was no difference in the distance traveled over time between the Tg and WT mice, indicating no hyperactivity. **d** Learning and spatial memory was assessed using the Morris water maze. Mice were trained on the location of the platform for 5 days. After training on day 5, mice were subject to a probe test where the platform was removed and time spent in each quadrant was measured. Next, the platform was moved to the opposite quadrant, and mice were trained for 5 additional days with a probe test at the end of day 10. **e** The average latency to platform discovery was recorded for each training day. Tg #2 mice showed a significant delay in learning the location of the platform on day 2 and 4 (left). This was not observed in Tg #1 mice (right). Neither Tg line showed reversal learning deficits. **f** Tg #2 but not Tg #1 mice spent significantly less time searching in the platform quadrant than their WT littermates during the first probe trial (Line 1: WT platform quadrant = 31.33 ± 2.78, empty quadrant = 22.89 ± 0.93. Tg #1 platform quadrant = 35.95 ± 2.50, empty quadrant = 21.35 ± 0.83. Line 2: WT platform quadrant = 34.28 ± 2.02, empty quadrant = 21.91 ± 0.67. Tg #2 platform quadrant = 26.23 ± 2.49, empty quadrant = 24.59 ± 0.83). **g** Tg mice performed just as well as controls during the probe trial and spent more time in the platform quadrant than the other quadrants (Line 1: WT platform quadrant = 40.00 ± 2.40, WT empty quadrant = 20.29 ± 0.82, WT old platform quadrant = 12.70 ± 1.86. Tg #1 platform quadrant = 39.08 ± 3.25, Tg #1 empty quadrant = 20.47 ± 1.11, Tg #1 old platform quadrant = 14.32 ± 1.85. Line 2: WT platform quadrant = 36.97 ± 2.09, WT empty quadrant = 21.27 ± 0.71, WT old platform quadrant = 13.41 ± 1.40. Tg #2 platform quadrant = 38.63 ± 3.25, Tg #2 empty quadrant = 20.56 ± 1.10, Tg #2 old platform quadrant = 13.00 ± 1.84). NS. not significant. *p < 0.05. See Table S3 for *n*’s

Anxiety is often reported in children with Dup15q and Fragile X [49, 50] and commonly comorbid in children with ASD [51]. To assess h*CYFIP1* Tg mice for anxiety, we tested animals in the elevated plus maze scored for time spent in the closed, protected arms versus the open, vulnerable arms [22]. Mice with increased anxiety spend more time in the closed arms compared control mice. h*CYFIP1* Tg mice did not differ from their control littermates in the time spent in the open and closed arms (Fig. 3a), indicating no increased anxiety. We further tested general anxiety using the open field test. We recorded and analyzed whether Tg mice spent excessive time along the edges of the field which indicates increased anxiety. Both lines spent similar time in the center and surround areas of the open field compared to their respective control littermates, indicating no significant anxiety (Fig. 3b). Additionally, we found no observable hyperactivity or motor dysfunction in either Tg line in the open field test as measured by distance traveled over time (Fig. 3c).

### CYFIP1-overexpressing mice display subtle learning and memory deficits and overt increased fear, but no deficits in sensory processing

We next sought to assess spatial learning and memory in h*CYFIP1* Tg mice using the Morris Water Maze (MWM; [52]). Mice were trained for 5 days with a probe trial on day 5 and then trained on the reversal for 5 more days with a second probe trial on day 10 (Fig. 3d). Tg line 2 mice demonstrated subtle learning deficits during the first 5 days of training (Fig. 3e, right), as well as during the first probe trial (Fig. 3f). However, these deficits were not present in Tg line 1. Since spatial learning and memory is a hippocampal dependent behavior [53], this difference may be caused by the lack of h*CYFIP1* protein overexpression in the hippocampus observed in line 1 (Fig. 1d). Both lines performed similar to their control littermates during reversal training and probe 2 (Fig. 3e and g). Overall, h*CYFIP1* mice demonstrate initial, subtle spatial learning deficits as measured by navigation in the Morris water maze, but eventually perform similar to their WT littermates, even when cognitive flexibility is challenged with a reversal task.

**Fig. 4:**
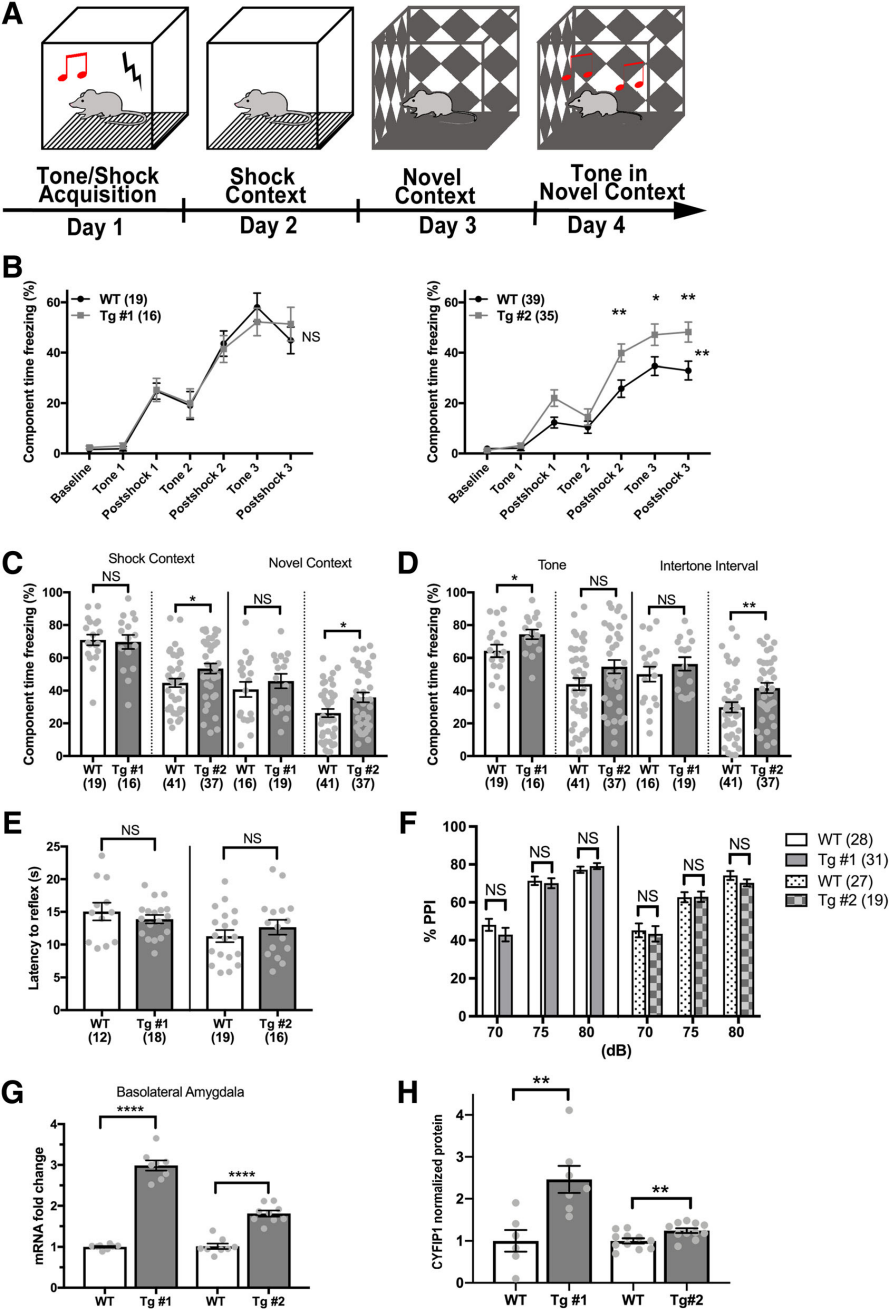
CYFIP1-overexpressing mice show increased fear in contextual and cued fear conditioning but do not demonstrate sensory processing deficits. **a** Schematic of fear conditioning protocol. Learning and memory of aversive stimuli was assessed using trace and contextual fear conditioning. **b** On day 1, freezing was recorded as mice were conditioned to a tone using a series of 3, 80-dB tones followed by 0.5-mA shocks. While all mice acquired the tone/shock association, there was a significant increase in freezing in the Tg #2 mice compared to their WT littermates during this acquisition phase. This increase in freezing was not observed in Tg #1 mice. **c** Tg #2 mice demonstrated an increase in freezing in the shock context which persisted when they were introduced to a novel context. There was no increase in freezing observed in the Tg #1 mice in the shock or novel contexts (Shock context: Line 1 WT = 70.91 ± 3.242, Tg #1, 69.69 ± 4.291. Line 2 WT = 44.78 ± 2.596, Tg #2, 53.54 ± 3.098. Novel context: Line 1 WT = 40.76 ± 4.613, Tg #1 = 45.89 ± 4.431. Line 2 WT, 26.27 ± 2.497, Tg #2, 35.87 ± 2.976). **d** Tg #1 mice showed a significant increase in freezing in response to the tone, and Tg #2 mice showed a significant increase in freezing in the intertone interval (Tone: Line 1 WT = 64.28 ± 3.793, Tg #1 = 74.37 ± 2.962. Line 2 WT = 44.04 ± 3.759, Tg #2, 54.68 ± 4.09. Intertone interval: Line 1 WT = 50.12 ± 4.585, Tg #1 = 56.38 ± 4.076. Line 2 WT = 29.84 ± 3.128, Tg #2, 41.67 ± 3.121). **e** The thermal pain reflex of Tg mice was assessed using the hot plate assay. The latency to paw withdrawal was similar in Tg and WT littermates (Line 1 WT = 15.05 ± 1.35, Tg #1 = 13.88 ± 0.65. Line 2 WT = 11.29 ± 0.92, Tg # 2 = 12.65 ± 1.13). **f** Sensory gating was assessed using the PPI test. Tg mice showed no differences in PPI at 70, 75, and 80 dBs compared to WT (70 dB: Line 1 WT = 48.11 ± 3.18, Tg #1 = 42.93 ± 3.57. Line 2 WT = 45.21 ± 3.63, Tg #2 = 43.40 ± 4.09. 75 dB: Line 1 WT = 71.37 ± 2.22, Tg #1 = 70.10 ± 2.65. Line 2 WT = 62.69 ± 2.66, Tg #2 = 62.97 ± 2.74. 80 dB: Line 1 WT = 77.20 ± 1.68, Tg #1 = 79.14. Line 2 WT = 74.19 ± 2.37, Tg #2 = 70.28 ± 1.83). CYFIP1 (**g**) mRNA and **h** protein levels in the basolateral amygdala were significantly increased in both Tg lines. NS, not significant. *p < 0.05. **p < 0.01. ***p < 0.001. ****p < 0.0001. See Additional file 3: Table S3 *n*’s

To assess fear in h*CYFIP1* overexpressing mice, a complex behavior involving the circuitry of the amygdala, hippocampus, and cortex [54–56], we performed contextual and cued fear conditioning. During tone-shock acquisition on day 1 (Fig. 4a), Tg line 2 mice froze significantly more than WT littermates after the second and third shocks, indicated a heightened fear response (Fig. 4b, right). This phenotype was not observed in Tg line 1 mice (Fig. 4b, left). Tg line 2 mice, but not Tg line 1 mice, also displayed an increase in freezing in the shock context 24 h after fear conditioning (Fig. 4c). Interestingly, this fear still persisted when placed in a novel environment 48-h post tone-shock acquisition (Fig. 4c), indicating an increase in generalized fear. Finally, both Tg lines demonstrated an increase in freezing to the conditioned tone in a novel environment 72 h after fear conditioning (Fig. 4d). Overall, while Tg line 2 mice show a robust response to fear conditioning in all parameters measured, there also was a fear response to the tone in Tg line 1 mice (Fig. 4d), indicating potential basolateral amygdala (BLA) dysfunction in h*CYFIP1* overexpressing animals. Since the circuits in the BLA are responsible for the tone association in fear conditioning [57], we performed qPCR and western blot analysis for h*CYFIP1* on acutely dissection BLA tissue, finding a significant increase in hCYFIP1 protein expression in both lines (Fig. 4g, h).

To differentiate the fear response in hCYFIP1 Tg mice from dysregulated sensory processing in which they perceived the stimuli as more painful, we examined the thermal pain reflex of Tg mice using the hotplate assay. There was no significant difference in the latency to reflex in either Tg line (Fig. 4e) indicating no increased sensation to thermal pain. We further investigated sensory processing in h*CYFIP1* mice using the prepulse inhibition (PPI) test. h*CYFIP1* mice showed no deficits in sensory gating when presented with pre-pulses at 70, 75, and 80 db (Fig. 4f). Therefore, while we do see differences in fear acquisition and behavior in *CYFIP1* Tg mice, we do not find any sensory processing deficits in our selected behavioral assays.

### RNA sequencing of the BLA reveals perturbations of genes related to GABAergic interneuron function, cytoskeletal organization, and myelination

**Fig. 5:**
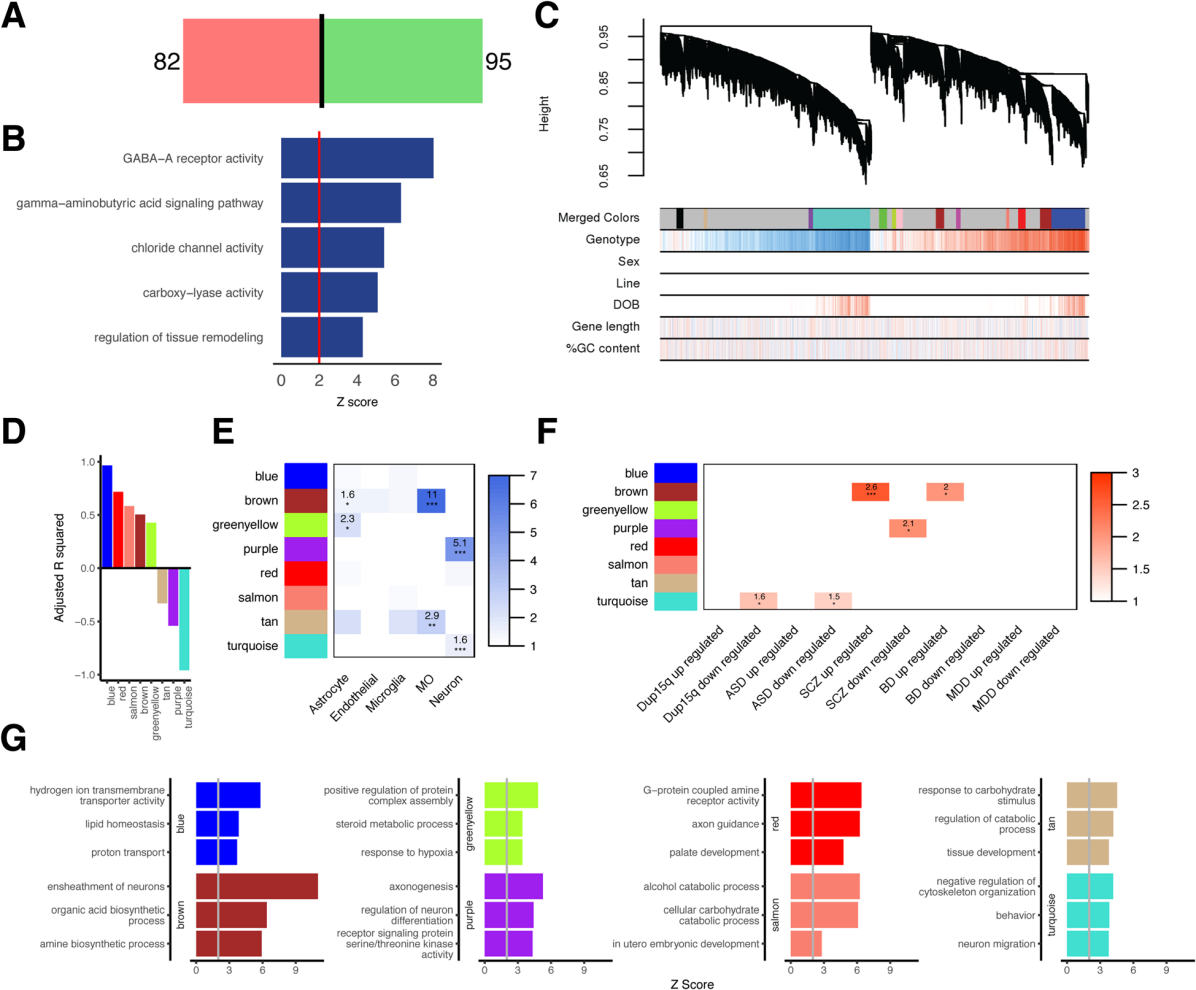
RNA sequencing of the BLA identifies molecular pathways underlying fear conditioning response, neuronal structure, and function. **a** Analysis of RNA sequencing from the BLA identified 177 differentially expressed genes (82 downregulated and 95 upregulated) when comparing CYFIP1 OE mice to their WT litter mates. **b** A list of the top 5 significant GO-terms derived from analysis of DE genes and their *Z*-scores. **c** Dendrogram produced from WGCNA analysis of the BLA transcriptome resulting in 13 modules. **d** *R-squared* values and directionality for the 8 modules significantly associated with genotype (fdr < 0.1), blue (*R*^2^ = 0.97), red (*R*^2^ = 0.72), salmon (*R*^2^ = 0.58), brown (*R*^2^ = 0.50), green-yellow (*R*^2^ = 0.43), tan (*R*^2^ = − 0.33), purple (*R*^2^ = − 0.54), and turquoise (*R*^2^ = − 0.96). **e** Cell type enrichment analysis for the significantly expressed modules. The numbers represent the odds ratio. **f** Enrichment of genes in each module associated with Dup15q, ASD, schizophrenia (SCZ), bipolar disorder (BD), and major depressive disorder (MDD). The numbers represent odds ratio. **g** A list of the top 3 significant GO-terms for each module and their *Z*-scores. *FDR < 0.05, **FDR < 0.01, ***FDR < 0.005

The fear conditioning phenotype observed in CYFIP1-overexpressing mice, led us to investigate the basolateral amygdala (BLA), an area of the amygdala that receives sensory input via the thalamus, cortex, and hippocampus and forms fear memories [58, 59]. To this end, we performed QuantSeq, a 3′ RNA sequencing technique (the “Methods” section), of the BLA in our h*CYFIP1* Tg mice. We identified 177 differentially expressed (DE) genes, 95 upregulated and 82 downregulated (Fig. 5a; Additional file 3: Table S4). The top 3 GO terms enriched in these DE genes, “GABA-A receptor activity,” “gamma-aminobutyric acid signaling pathway,” and “chloride channel activity” were all related to GABA receptor activity (Fig. 5b, Additional file 3: Table S5).

To move beyond analysis of single genes to investigate more coordinated transcriptional regulation, we performed weighted gene co-expression network analysis (WGCNA). We identified 8 modules significantly associated with genotype (out of a total of 13 modules; Fig. 5c), of which five modules were upregulated and three downregulated (Fig. 5d). The top GO terms in each BLA module highlight biologically functional roles for CYFIP1 in ATP synthesis (blue), myelination (brown), protein complex assembly (green-yellow), axonogenesis and neuronal differentiation (purple), axon guidance (red), alcohol catabolic process (salmon), response to carbohydrate stimulus (tan), and negative regulation of cytoskeleton organization (turquoise) (Fig. 5g). Cell type enrichment analysis using cell type-specific markers from mouse brain [40] reveals that the upregulated brown and green-yellow modules are enriched for astrocyte markers, the brown and tan modules are enriched for myelinating oligodendrocyte markers, and the downregulated purple and turquoise modules are enriched for neuronal markers (Fig. 5e). To test whether these modules were relevant to psychiatric disorders we tested for enrichment of these modules for genes found to be differentially expressed in post-mortem brain from patients with Dup15q, ASD, SCZ, BP, and MDD [60]. The downregulated turquoise module was enriched for genes that are downregulated in Dup15q and ASD, the downregulated purple module was enriched for genes that are downregulated in schizophrenia, and the upregulated brown module was enriched for genes that are upregulated in SCZ and BD (Fig. 5f). Overall, RNA sequencing of the BLA highlights a role for CYFIP1 in GABAergic subunit regulation, solidifies CYFIP1’s prominent role in regulation of cytoskeleton, and strengthens CYFIP1’s newly discovered role in myelination [61, 62]. Also, overlap of some of the modules with the gene expression signatures of those found in schizophrenia, bipolar disorder, Dup15q, and ASD, demonstrating that the effect of CYFIP1 overexpression affects pathways observed across several psychiatric disorders.

## Discussion

We investigated the effects CYFIP1 overexpression on rodent behavior and neuronal gene expression by overexpressing human CYFIP1 in mice. These mice did not display any changes in the core behavioral phenotypes of ASD, social behavior, and repetitive, restrictive behaviors, suggesting that CYFIP1 overexpression may not be a major factor in these phenotypes. However, CYFIP1 overexpression is not without consequences. We did detect more subtle behavioral phenotypes such as learning and memory deficits and increased fear conditioning. This is consistent with large clinical databases which find that lone *CYFIP1* duplication is present in 0.5–1% of subjects, most of whom are neurotypical, and suggests that it is some combination of the 3 other genes, *NIPA1*, *NIPA2*, and *TUBGCP5*, in the duplication region or even unidentified genetic mutations outside the region that contribute to the severe, but variable, ASD-related phenotypes observed in humans with BP1-2 dup [12, 63–66].

Our mouse behavioral phenotypes are echoed in research describing patients with BP1-2 duplications (which include the CYFIP1 gene), noting that while half of BP1-2 duplication carries have developmental delay or speech delay [63], the phenotypes are highly variable, signifying that additional, unknown genetic modifiers may be necessary to cause ASD-associated deficits [12, 64]. Perhaps the most remarkable behavioral phenotype found in this study is the overt increase in conditioned fear. Interestingly, neither line demonstrated increased anxiety in the open field or elevated plus maze, signifying that the phenotype found in the fear conditioning assay is solely indicative of fear learning and not generalized anxiety. Fear is often observed in Dup15q mice [67] and children with fragile X syndrome [68–70], caused by mutations in FMRP which is a known CYFIP1-interacting protein. Overall, it is reasonable to conclude that overexpression of a CYFIP1, a single gene duplicated in Dup15q syndrome, results in subtle yet significant behavioral phenotypes that encompass a specific aspect of the greater syndrome.

To characterize the molecular pathways underlying the increase in fear demonstrated in CYFIP1 OE mice, we performed RNA sequencing of the BLA and found increases in gene expression of many GABA related genes such as *Nova1*, *Nkx2*.*1*, *Calb1*, as well as the GABA-A receptor subunit genes: *Gabra1*, *Gabrg1*, and *Gabrg2* which are expressed by PV-interneurons in the basolateral amygdala [71]. While a decrease in GABA-A receptor subunits has been observed in the cortex of FXS knockout mice [72], a direct connection between CYFIP1 expression and inhibitory synaptic structure and function is just beginning to be explored [73]. PV-expressing GABAergic neurons in the BLA are important for the integration and neuromodulation of fear memory formation [74], and perturbations of this tightly regulated circuit may be responsible for the increase in fear conditioning that we observe in our CYFIP1 overexpressing mice. During fear conditioning, these PV-interneurons are excited and concurrently inhibit the principal neurons of the BLA, which are essential for integration of the conditioned stimulus with fear memory formation (for an in-depth review, see [74]). Therefore, it is possible that the increase in expression of these receptor subunits of CYFIP1 OE mice are responsible for the observed exaggerated fear response.

CYFIP1’s known molecular functions include roles in spine formation [14], neurite outgrowth [16], and axonal outgrowth [75, 76], all mediated via CYFIP1’s membership in the WAVE regulatory complex (WRC) which regulates F-actin assembly and disassembly. While many of the GO terms from our sequencing analysis contain biological processes in agreement with CYFIP1’s canonical functions; surprisingly, the genes underlying these GO-terms do not belong to members of the WRC or its known binding partners. This is surprising because it was previously hypothesized that depletion of CYFIP1 expression affected mRNA expression of Nap1, Abi1, Wave1, and HSP300, all members of the WRC [18]. Our data from the highly neuronally-enriched BLA turquoise module reveals GO-terms related to regulation of cytoskeleton organization. However, many of the genes in this module (i.e., APC, Shank1, Shroom2, Tmsb10) function in the recruitment the WRC to the cytoskeleton [77], rather than via transcriptional regulation of WRC members. This finding highlights a potential role for CYFIP1 in regulating the proteins necessary for trafficking and recruiting WRC member proteins to the cytoskeleton as opposed to directly regulating the translation of WRC member proteins as previously hypothesized [15]. In summary, our RNA sequencing analysis of CYFIP1 overexpression not only provides confirmation of known CYFIP1 function, but also suggests novel molecular pathways through which this regulation is accomplished such as recruitment of WRC to the cytoskeleton.

Another intriguing finding from our sequencing analysis is the effect of CYFIP1 overexpression on myelination. The top GO-term in the brown module, “ensheathment of neurons” contains the genes *Mbp*, *Plpl1*, and *Cldn11* which are all key components of myelin. Significant enrichment for genes expressed by myelinating oligodendrocytes two of the BLA modules (brown and tan) is intriguing since oligodendrocyte dysfunction has been linked to both autism and schizophrenia [78–81] but has yet to be described in Dup15q syndrome. While CYFIP1’s direct role in myelination is just beginning to be examined [62], WAVE1’s role in oligodendrocyte morphogenesis is well established [82–85]. Further, the WAVE complex regulates ARP2/3 which is necessary for initiation of myelination due to its role in actin filament assembly [86], and Cdc42 and Rac1 signaling, both affected by the WAVE complex, and are also necessary for myelin sheath formation [87]. Overall, our RNA sequencing data and other studies suggest that CYFIP1 overexpression affects myelination and oligodendrocyte number and maturation [62]. This is most likely mediated via CYFIP1’s role in the WAVE complex and subsequent downstream signaling demonstrated by the previously identified importance of this pathway in myelination.

Abnormal CYFIP1 expression levels are often observed as part of larger, multi-genetic syndromes. Therefore, it is important to elucidate which molecular and behavioral phenotypes are solely due to this single gene dysfunction as shown here and which disease traits are due to a complex combination of genetic factors. The evidence from two mouse lines overexpressing human CYFIP1 does not support that CYFIP1 overexpression leads to ASD-like behaviors in this mouse model. These mice are normal in the vast majority of behavioral tests utilized in this study. This suggests other genes in the BP1-2 region are responsible or that there is other unknown genetic susceptibility that when combined with CYFIP1 duplication results in disease. However, there are subtle behavioral deficits, importantly and most specifically in fear conditioning which is mirrored by changes in gene expression in the BLA. By understanding the transcriptional consequences that are perturbed by these single-gene changes, we can begin to unravel the underlying pathways that are perturbed in complex, genetic disorders such as ASD.

## Additional files

Additional file 1: **Figure S1.** Protein analysis of CYFIP1 at p21. There was a significant increase in CYFIP1 expression in the cortex of mice at p21 in line 1 (WT = 1 ± 0.08, Tg#1 = 1.97 ± 0.32) as well as line 2 (WT = 1 ± 0.05, Tg#2 = 1.45 ± 0.13). **p < 0.001. See Table S1 for n’s. (TIF 84 kb)

Additional file 2: **Figure S2.** Timeline representing order of behavioral tests and ages at which they were performed. HCB home-cage behavior, OFT open field test, 3CS three-chamber social test, HP hotplate, EPM elevated plus maze, FC fear conditioning, MWM Morris water maze, PPI prepulse inhibition. (TIF 3934 kb)

Additional file 3: **Table S1.** Number of animals for molecular characterization of CYFIP1 OE Mice. **Table S2.** SHIRPA scoring criteria and results. **Table S3.** Mouse age, sex, number, and statistics used for behavioral experiments. **Table S4.** List of differentially expressed genes from RNA sequencing of the basolateral amygdala. **Table S5.** WGCNA GO-term enrichment for RNA sequencing of the basolateral amygdala. (XLSX 82 kb)

## Data Availability

All behavioral data generated and analyzed during this study are included in this published article and its supplementary information tables. The RNA seq datasets generated and analyzed during the current study are available at GSE125697.

## Abbreviations

ASD: Autism spectrum disorder
BAC: Bacterial artificial chromosome
BLA: Basolateral amygdala
BPs: Breakpoints
CNVs: Copy number variations
CYFIP1: Cytoplasmic FMRP-interacting protein 1
DE: Differentially expressed
Dup15q: 15q duplication syndrome
hCYFIP1: Human-CYFIP1
MWM: Morris water maze
P#: Postnatal day #
PPI: Prepulse inhibition
Tg: Transgenic
USVs: Ultrasonic vocalizations
WGCNA: Weighted gene co-expression network analysis
WRC: Wave regulatory complex
WT: Wild-type
