# Supplementary figures and images for: CYFIP1 overexpression increases fear response in mice but does not affect social or repetitive behavioral phenotypes
Source: Mol Autism. 2019 Jun 7;10:25. doi: 10.1186/s13229-019-0278-0 (PMC6555997; doi:10.1186/s13229-019-0278-0)

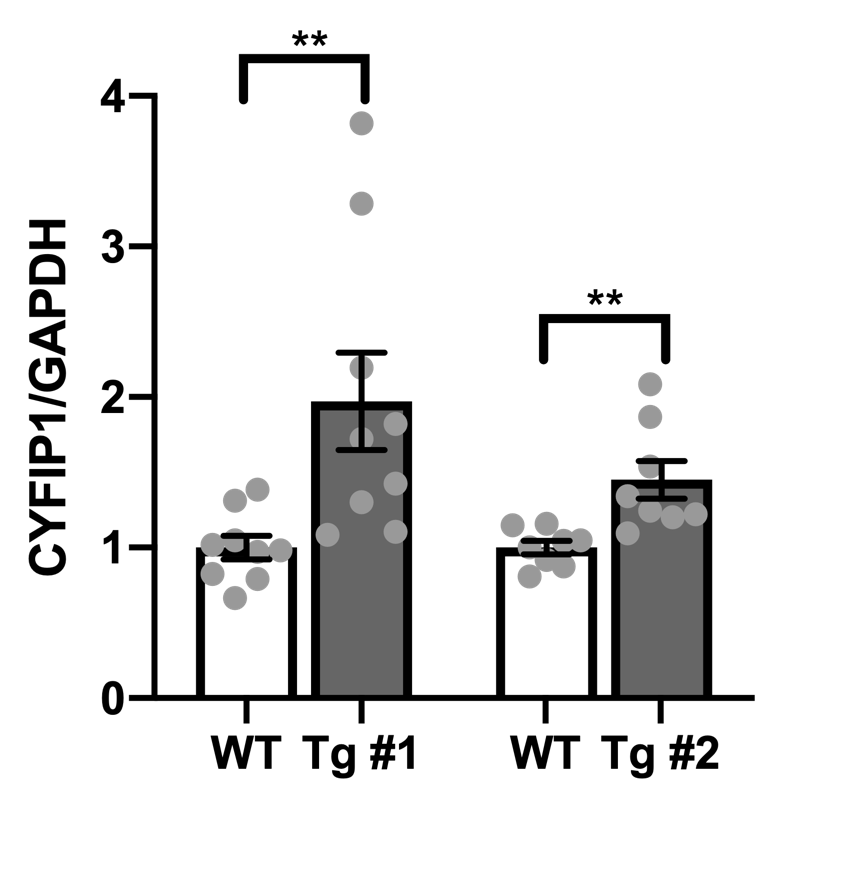

Supplement: Supplementary file 1 — Figure S1. Protein analysis of CYFIP1 at p21. There was a significant increase in CYFIP1 expression in the cortex of mice at p21 in line 1 (WT = 1 ± 0.08, Tg#1 = 1.97 ± 0.32) as well as line 2 (WT = 1 ± 0.05, Tg#2 = 1.45 ± 0.13). **p < 0.001. See Table S1 for n’s. (TIF 84 kb) [file 13229_2019_278_MOESM1_ESM.tif]

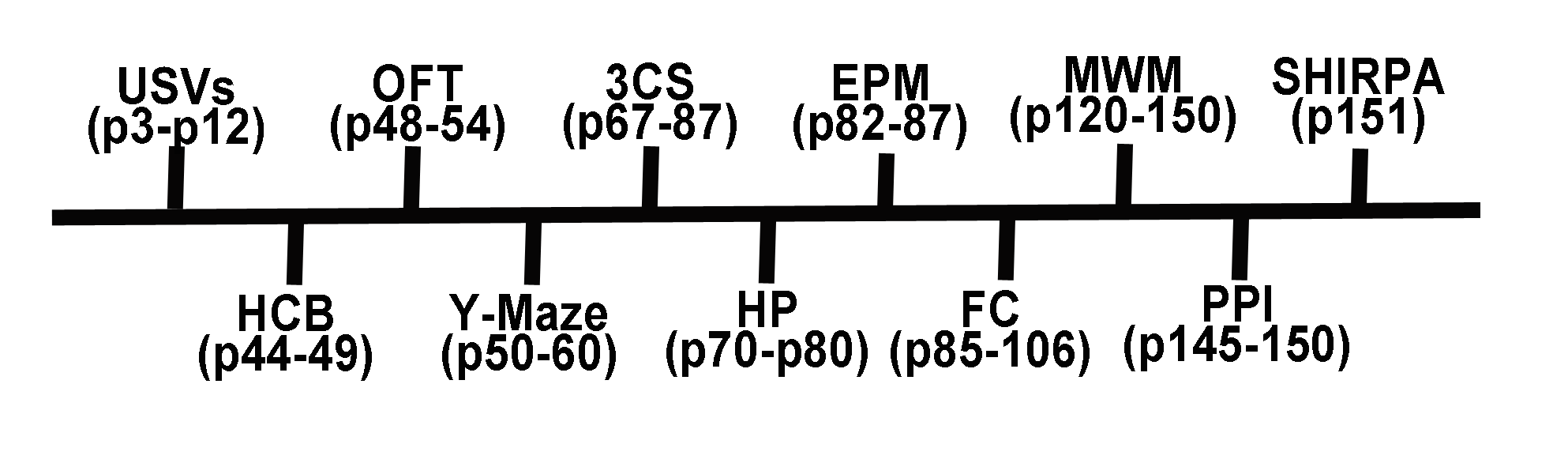

Supplement: Supplementary file 2 — Figure S2. Timeline representing order of behavioral tests and ages at which they were performed. HCB home-cage behavior, OFT open field test, 3CS three-chamber social test, HP hotplate, EPM elevated plus maze, FC fear conditioning, MWM Morris water maze, PPI prepulse inhibition. (TIF 3934 kb) [file 13229_2019_278_MOESM2_ESM.tif]
